# The Potential Renal Protective Effect of Transcatheter Aortic Valve Replacement

**DOI:** 10.7759/cureus.80465

**Published:** 2025-03-12

**Authors:** Deepa Soodi, Paul Yeung, Peter Umukoro, Somto T Nwaedozie, Rachel Gabor, Eric DeJarlaris, Param Sharma, Romel Garcia-Montilla

**Affiliations:** 1 Cardiology, Marshfield Clinic Health System, Marshfield, USA; 2 Internal Medicine, Marshfield Medical Center, Marshfield, USA; 3 Nephrology, Hendricks Regional Health, Danville, USA; 4 Internal Medicine, Marshfield Clinic Health System, Marshfield, USA; 5 Research, Marshfield Medical Center, Marshfield, USA; 6 Critical Care Medicine, Marshfield Clinic Health System, Marshfield, USA

**Keywords:** acute kidney injury, aortic stenosis, chronic kidney disease, renal failure, tavr

## Abstract

Background

The incidence of aortic stenosis (AS) is steadily increasing, posing a significant healthcare burden. Transcatheter aortic valve replacement (TAVR) is being used more frequently to treat patients with symptomatic AS. This study evaluated long-term changes in renal function and mortality in TAVR patients over a period of up to three years, including those with normal creatinine (Cr) levels and those with chronic kidney disease (CKD).

Methods

We conducted a retrospective review of 270 patients who underwent TAVR between 2012 and 2017 at a rural tertiary referral center. Collected data included baseline serum Cr and estimated glomerular filtration rate (eGFR), with follow-up measurements taken at 30 days, six months, one year, two years, and three years post-TAVR. Patients were categorized into two groups: those with CKD and those without.

Results

Both groups showed similar improvements in eGFR at one month (6.3 mL/min/m², p < 0.001). However, by three months, eGFR levels returned to their pre-TAVR baseline. At the three-year mark, an average decline of 5.3 mL/min/m² was observed in both groups (p < 0.001). Despite CKD patients having worse kidney function throughout the study period, the extent of eGFR reduction was similar between the CKD and non-CKD groups, indicating that eGFR decline was independent of CKD status. Mortality rates were higher in CKD patients (56.9 (39%) vs. 24.6 (22%); p = 0.006). Multivariate analysis identified CKD as the most reliable predictor of mortality.

Conclusions

Renal function significantly improved at one month post-TAVR in both CKD and non-CKD patients. Although eGFR initially improved after TAVR, the subsequent decline was similar in both groups, suggesting that the reduction in eGFR is independent of CKD status. Cardiorenal syndrome, which can occur with AS, may improve with TAVR. These findings support the potential renoprotective effect of TAVR in patients with CKD.

## Introduction

Aortic stenosis (AS) is the most common age-related acquired valvular disease, with a prevalence of 1.3% in individuals aged 65-74 years, 4.6% in those over 75 years, and 8% in people aged 85 years or older [1]. The incidence of AS continues to rise due to an aging population, making it a significant healthcare burden [2]. Without treatment, patients with severe symptomatic AS face a 50% mortality rate within the first two years [3].

Surgical aortic valve replacement (SAVR) remains the gold standard for treating severe symptomatic AS. However, transcatheter aortic valve replacement (TAVR) has emerged as a viable alternative, particularly for patients at prohibitive surgical risk due to comorbidities and high surgical risk [4]. Prohibitive risk is defined by the presence of at least one of the following Society of Thoracic Surgeons predicted risks: postoperative mortality ≥8%, severe aortic calcification such as porcelain aorta, severe frailty, hostile chest anatomy, severe liver disease (Model for End-Stage Liver Disease score >12), severe pulmonary hypertension, complex anatomy (e.g., internal mammary grafts at risk of injury), increased bleeding diathesis, chemotherapy for malignancy, immobility, severe dementia, or acquired immunodeficiency syndrome [5].

Due to these risk selection criteria, patients undergoing TAVR are generally older and have a higher prevalence of chronic kidney disease (CKD) compared to those receiving SAVR [6]. Multiple studies have demonstrated the negative impact of impaired kidney function and acute kidney injury (AKI) on mortality rates following cardiac surgery [7-9]. According to a study by Voigtländer et al. [5], patients at high surgical risk who underwent TAVR experienced greater 12-month survival rates compared to those who underwent SAVR.

Kidney function, particularly the occurrence of AKI, significantly affects patient outcomes after TAVR, as highlighted by recent research [10,11]. The updated logistic EuroSCORE [12] and Valve Academic Research Consortium criteria [13] emphasize the importance of renal function when assessing the risk of severe AS in the general population and AS patients, respectively.

Given this background, the purpose of the present study was to assess the impact of TAVR on renal function, measured by serum creatinine (SCr) levels and estimated glomerular filtration rate (eGFR), over a three-year period following the procedure.

We propose that renal function may initially decline following TAVR due to intraoperative factors such as hypotension during rapid pacing and embolization. However, we anticipate improvement in kidney function over six months and beyond, driven by enhanced renal perfusion resulting from improved cardiorenal syndrome (CRS) after TAVR.

Additionally, one of our hypotheses is that patients with preexisting, non-dialysis CKD will experience worse kidney outcomes compared to those without any preexisting kidney disorders. This difference is expected due to altered baseline kidney function in CKD patients, with worsening kidney function potentially representing an acute-on-chronic decline.

This article abstract was previously presented at the ACC 2022 Scientific Meeting on March 8, 2022.

## Materials and methods

Study design

This retrospective study was conducted at a tertiary referral center in central Wisconsin, USA, including 270 patients who underwent TAVR procedures between January 1, 2012, and December 31, 2017. Patients who underwent TAVR at other facilities and those on dialysis were excluded. The renal function dataset was extracted from the electronic health records of Marshfield Clinic Health System (MCHS) using ICD-9/10 codes for TAVR. CKD was defined as kidney damage or reduced kidney function lasting three or more months, regardless of the cause. eGFR was calculated using the Modification of Diet in Renal Disease formula. The study received approval from the Institutional Review Board (approval number IRB00000673).

Data collection

Data abstraction was performed by electronically extracting relevant data from the MCHS electronic health records. To ensure accuracy, 10% of the electronically retrieved data was manually verified by the principal investigators. In addition to the clinical parameters mentioned above, SCr and eGFR were measured at 30 days, six months, one year, two years, and three years post-TAVR.

Statistical analysis

Baseline characteristics were summarized using means and SDs for continuous variables and counts and percentages for categorical variables. Comparisons between patients with CKD and those without at the time of TAVR were conducted using t-tests or chi-squared tests, as appropriate.

Patient follow-up data, including mortality, medication use, and kidney function, were reported at various intervals over the three years following TAVR. SCr and eGFR were described using means (SD), and short-term changes in eGFR were assessed using paired t-tests.

Long-term trends in eGFR were analyzed using a linear mixed model. Linear and nonlinear time effects, random effects, and variance structures were evaluated using root mean square error (RMSE). The generalized additive mixed model, which incorporated random intercepts for each patient and allowed different variance structures based on baseline CKD, demonstrated the lowest RMSE.

Interactions between CKD, time, and angiotensin-converting enzyme inhibitor (ACEi) or angiotensin receptor blocker (ARB) use were tested but found to be insignificant. The final model was adjusted for patient demographics, baseline medication use, and comorbidities, excluding hyper- or dyslipidemia due to high collinearity with hypertension. Model fit was evaluated using residual plots.

Median survival time was calculated, and an adjusted Cox proportional hazards model was developed to identify risk factors for mortality following TAVR. Covariates included demographics, baseline medication use, and comorbidities, excluding hyper- or dyslipidemia. Model fit was assessed using Schoenfeld and Martingale residuals, and adjusted survival curves by CKD status were generated.

All statistical analyses were performed using R version 4.0.2, with packages including ggplot2, caret, nlme, splines, mgcv, itsadug, survival, and survminer.

## Results

Patient population and baseline characteristics

Baseline characteristics were collected from patients before undergoing TAVR, including age, sex, comorbid conditions (such as hypertension, diabetes mellitus, congestive heart failure (CHF), coronary artery disease, and dyslipidemia), and medication use (including ACEi and ARBs).

Kidney function was assessed by measuring baseline eGFR and SCr levels for both the CKD and non-CKD groups. A summary of the baseline demographics and clinical characteristics for each group is provided in Table 1.

**Table 1:** Patient demographics, comorbidities, and medication use at TAVR by CKD Means and SDs are reported for continuous variables, with t-tests used to compare means based on CKD diagnosis at baseline. For dichotomous variables, counts and percentages are provided, and chi-squared tests are used to compare distributions between the CKD and non-CKD groups at baseline. ACEi, angiotensin-converting enzyme inhibitor; ARB, angiotensin receptor blocker; CAD, coronary artery disease; CHF, congestive heart failure; CKD, chronic kidney disease; eGFR, estimated glomerular filtration rate; SCr, serum creatinine; TAVR, transcatheter aortic valve replacement

Characteristic	All (N = 258)	No CKD at TAVR (N = 112)	CKD at TAVR (N = 146)	p-Value
Demographics and operative details				
Age at TAVR (mean ± SD)	82 (7.4)	81.1 (8.4)	82.8 (6.6)	0.08
Sex				
Female (%)	130 (50.4)	54 (48.2)	76 (52.1)	0.627
Male (%)	128 (49.6)	58 (51.8)	70 (47.9)	
Valve type				
Balloon expandable (%)	178 (69.0)	81 (72.3)	97 (66.4)	0.381
Self expandable (%)	80 (31.0)	31 (27.7)	49 (33.6)	
Comorbidities				
Hypertension (%)	242 (93.8)	98 (87.5)	144 (98.6)	<0.001
Diabetes (%)	121 (46.9)	41 (36.6)	80 (54.8)	0.006
CHF (%)	200 (77.5)	69 (61.6)	131 (89.7)	<0.001
CAD (%)	229 (88.8)	96 (85.7)	133 (91.1)	0.247
Hyper- or dyslipidemia (%)	227 (88.0)	92 (82.1)	135 (92.5)	0.02
Medication use				
ACEi use (%)	99 (38.4)	46 (41.1)	53 (36.3)	0.515
ARB use (%)	52 (20.2)	16 (14.3)	36 (24.7)	0.057
Kidney function				
Baseline eGFR (mL/min/1.73m²)	57 (16.1)	69.4 (11.0)	47.5 (12.7)	<0.001
Baseline SCr (mg/dL)	1.2 (0.4)	0.9 (0.2)	1.4 (0.4)	<0.001

The average age of patients undergoing TAVR was 82 years (SD: 7.4). Among the 258 patients, 242 (94%) had hypertension, and 121 (46.9%) had diabetes mellitus. Of the total patients, 112 did not have CKD at the time of TAVR, while 146 did. Additionally, 14 patients (12.5%) were diagnosed with CKD within three years after TAVR; however, they were kept in their original non-CKD group for analysis (Effect estimates from the multivariate longitudinal eGFR model).

eGFR trends following TAVR

Table 2 presents the baseline eGFR and SCr levels measured at one, three, and six months, as well as one, two, and three years post-TAVR. The average baseline eGFR for both groups was 57.0 mL/min/m², which improved to 63.5 mL/min/m² at one month post-TAVR. Both groups experienced a similar improvement in eGFR at one month, with an average increase of 6.3 mL/min/m² from baseline. This improvement was statistically significant (p < 0.001).

**Table 2:** Kidney function, medications, and patient counts during the three-year follow-up SCr and eGFR are reported as means (SD). Patients taking medications are reported as counts (%). Patients with no additional follow-up and deceased patients are reported as counts (%). ACE, angiotensin-converting enzyme; ARB, angiotensin receptor blocker; eGFR, estimated glomerular filtration rate; SCr, serum creatinine

Category	Baseline	One month	Three months	Six months	One year	Two years	Three years
Kidney function							
eGFR	57.0 (16.9)	63.5 (17.9)	52.9 (17.7)	51.4 (15.9)	54.3 (17.0)	51.2 (14.8)	51.4 (17.4)
SCr	1.2 (0.4)	1.1 (0.4)	1.3 (0.6)	1.3 (0.6)	1.3 (0.6)	1.2 (0.4)	1.3 (0.5)
Medications							
ACEs	99 (38.4%)	86 (33.3%)	53 (20.5%)	40 (15.5%)	44 (17.1%)	32 (12.4%)	23 (8.9%)
ARBs	52 (20.2%)	48 (18.6%)	32 (12.4%)	32 (12.4%)	33 (12.8%)	21 (8.1%)	18 (7.0%)
Patient counts							
Deceased (cumulative)	0 (0%)	5 (1.9%)	16 (6.2%)	22 (8.5%)	34 (13.2%)	55 (21.3%)	82 (31.8%)
No additional follow-up	0 (0%)	8 (3.1%)	24 (9.3%)	26 (10.1%)	46 (17.8%)	92 (35.7%)	-

**Figure 1:**
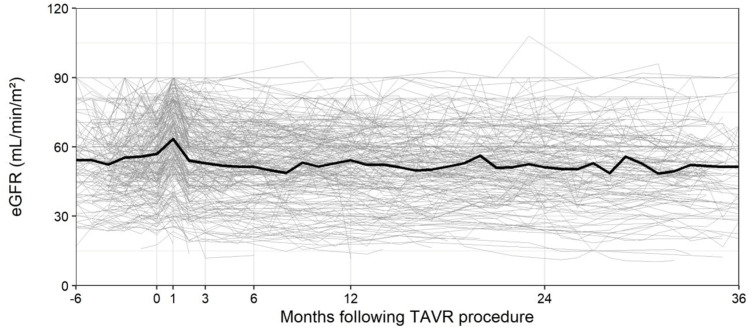
Average eGFR following TAVR eGFR, estimated glomerular filtration rate; TAVR, transcatheter aortic valve replacement

**Figure 2:**
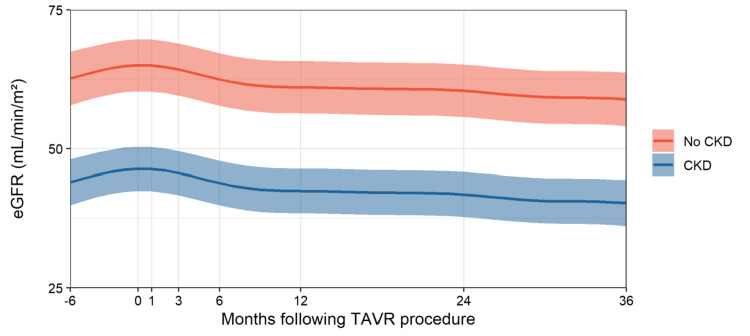
Estimated eGFR trends following TAVR procedure in CKD and non-CKD groups CKD, chronic kidney disease; eGFR, estimated glomerular filtration rate; TAVR, transcatheter aortic valve replacement

However, by three months post-TAVR, the eGFR had returned to baseline levels (Figure 1, Figure 2). At the three-year mark, an average decrease of 5.3 mL/min/m² in eGFR was observed across both groups (p < 0.001). Despite patients with CKD consistently showing worse kidney function at all points, the rate of eGFR decline over time was similar between the CKD and non-CKD groups (Figure 2).

A significant longitudinal trend in eGFR following TAVR was identified (p < 0.001), with the decline being nonlinear, as illustrated in Figure 2. Sex was a significant predictor of eGFR, with men having eGFR values approximately 3.8 units higher than women (95% CI: 0.8-6.7). Age also played a significant role, with eGFR decreasing by approximately 0.3 units (95% CI: -0.5 to -0.1) for each additional year of age.

After adjusting for confounders, CKD remained a significant predictor of eGFR. On average, patients with CKD had eGFR values 18.6 mL/min/m² lower than those without CKD (95% CI: -21.9 to -15.4, p < 0.001). The interaction between time and CKD was not significant (p = 0.330), nor were the interactions between time and ACE inhibitor use (p = 0.297) or ARB use (p = 0.274).

Differences in long-term eGFR trends following TAVR by CKD

Patients without CKD had a baseline eGFR of 69 mL/min/m², which declined to 63 mL/min/m² over the three-year period following TAVR. In contrast, patients with CKD had a baseline eGFR of 47.6 mL/min/m², which decreased to 43.2 mL/min/m² three years post-TAVR (Table 3).

**Table 3:** Kidney function following TAVR by CKD Kidney function after TAVR in patients with CKD was assessed using SCr and eGFR, reported as means (SD). CKD status was considered a time-varying variable. CKD, chronic kidney disease; eGFR, estimated glomerular filtration rate; SCr, serum creatinine; TAVR, transcatheter aortic valve replacement

Group	Baseline	One month	Three months	Six months	One year	Two years	Three years
No CKD							
eGFR	69.0 (12.0)	76.5 (11.1)	65.6 (12.4)	62.0 (7.7)	63.8 (14.8)	59.7 (13.8)	63.0 (14.9)
SCr	0.9 (0.2)	0.8 (0.2)	1.0 (0.2)	1.0 (0.2)	1.1 (0.7)	1.0 (0.3)	1.1 (0.5)
CKD							
eGFR	47.6 (14.0)	53.5 (15.6)	45.8 (16.1)	46.2 (16.4)	46.3 (14.6)	43.9 (11.5)	43.2 (14.2)
SCr	1.4 (0.4)	1.3 (0.4)	1.5 (0.7)	1.5 (0.6)	1.4 (0.5)	1.4 (0.4)	1.5 (0.5)

At baseline, significant differences were observed between the groups regarding hypertension, diabetes, CHF, and hyper- or dyslipidemia. Age and ARB use at the time of TAVR were marginally significant. Additionally, the proportion of patients who died within three years was notably higher in the CKD group (39%, n = 56.9) compared to the non-CKD group (22%, n = 24.6), with a p-value of 0.006. However, TAVR-related mortality did not differ significantly between the two groups.

Survival analysis

**Figure 3:**
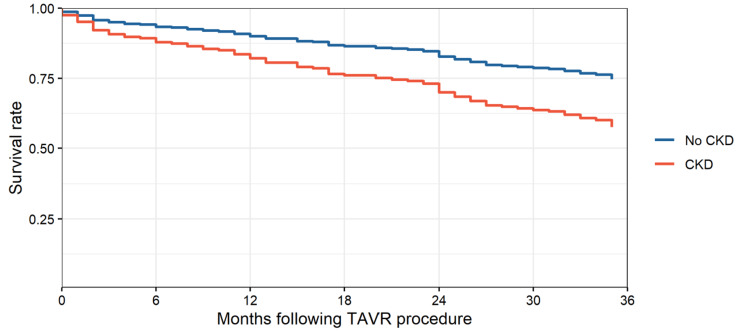
Adjusted survival curves by baseline CKD following TAVR procedure CKD, chronic kidney disease; TAVR, transcatheter aortic valve replacement

We used the Cox proportional hazards model and excluded hyper- or dyslipidemia due to strong collinearity with hypertension. While hypertension was significant at the univariate level, hyper- or dyslipidemia was not. Diabetes was included in the model because of its protective effect, and since all hypertensive patients also had diabetes, although diabetes was not significant at the univariate level. HRs were obtained from our adjusted Cox model. CKD increased the hazard of death by a factor of 1.74 (p = 0.045), and hypertension increased the hazard by a factor of 2.51 (p = 0.063). For men, the hazard of death increased by a factor of 1.42 (p = 0.077), and CHF increased the hazard by a factor of 1.60 (p = 0.094) (Table 4). The proportion of patients who died within three years was significantly higher in the CKD group compared to the non-CKD group (CKD group: 56.9% vs. non-CKD group: 24.6%; p = 0.006) (Figure 3). A total of 82 patients (31.8% of the study population) died within three years, with patients with CKD experiencing a higher mortality rate (56.9% vs. 24.6%; p = 0.006).

**Table 4:** HR estimates from adjusted Cox proportional hazards model ACE, angiotensin-converting enzyme; ARB, angiotensin receptor blocker; CAD, coronary artery disease; CHF, congestive heart failure; CKD, chronic kidney disease; eGFR, estimated glomerular filtration rate

Variable	HR	95% CI	p-Value
Age	1.00	0.97-1.03	0.837
Male	1.42	0.97-2.09	0.077
Baseline eGFR	1.00	0.98-1.02	0.992
CKD	1.74	1.13-2.98	0.045
Self-expandable (reference: balloon expandable)	1.02	0.69-1.52	0.915
Hypertension	2.51	0.96-6.60	0.063
Diabetes	0.71	0.46-1.09	0.113
CHF	1.60	0.92-2.77	0.094
CAD	1.16	0.57-2.36	0.674
Baseline ACE use	0.91	0.60-1.38	0.657
Baseline ARB use	0.66	0.39-1.09	0.106

## Discussion

The incidence of AKI leading to CKD after cardiac surgery ranges from 5% to 10%, but it can rise up to 25% following SAVR. Several risk factors, including hypertension, chronic obstructive pulmonary disease, thrombocytopenia, leukocytosis, and CKD, are associated with this progression [14]. TAVR patients are typically older and have higher rates of comorbid conditions [15], such as hypertension, diabetes mellitus, and CKD, which may increase the risk of AKI [16]. We suspect that our patient population was sicker than those in previously reported studies [17]. Procedural complications - such as intraoperative contrast exposure, hypotension during rapid pacing, arterial microembolization, valve debris dislodgement during deployment, and bleeding - can lead to AKI in up to 50% of TAVR cases [18].

On the other hand, improvements in hemodynamics after TAVR, such as enhanced cardiac output, reduced vascular tone, and better renal perfusion, could slow the progression of CKD. According to Goebel et al. [19], the amount of contrast used during the procedure does not affect the risk of AKI. The hemodynamic effects of AS may have a similar impact on kidney function, particularly in patients with CHF and type II CRS. Moreover, a similar link between heart failure and renal function has been established, where deteriorating renal function accelerates the progression of AS, initiating a vicious cycle of worsening organ dysfunction. Aortic valve replacement can potentially break this cycle by improving cardiac output. Our study focused on the effect of TAVR on kidney function. While the precise reasons for increased morbidity and mortality after TAVR in patients with renal dysfunction remain unclear, they are likely multifactorial and related to the baseline high-risk profile of this population.

Combined data from the PARTNER1 and PARTNER2 trials indicated that, in patients with intermediate or high surgical risk undergoing TAVR with a balloon-expandable valve, CKD stages improved or remained stable in 89% of patients seven days post-TAVR, with a negligible risk of CKD progression (0.035%) [20]. Although we did not categorize patients by valve type, we observed a similar improvement in eGFR in both groups at one month, with an average change in eGFR from baseline to one month of 6.3 mL/min/m². Witberg et al. [21] conducted a retrospective cohort study that included 894 patients who underwent TAVR. They found that kidney function improved or remained stable in 80.6% of patients (with improvement in 36.8%) one month after TAVR. Additionally, they analyzed survival according to CKD status pre- and post-TAVR and found that patients whose CKD status improved after TAVR had similar survival rates to those with no baseline CKD. Beohar et al. [20] analyzed data from the PARTNER 1 trial and registry, which showed that worsening renal function after TAVR in patients with baseline CKD was associated with increased one-year mortality. In other trials, nearly 50% of patients in CKD stage IV showed improvement in their functional stage following TAVR, and fewer than 1% progressed to CKD stage V.

While we did not subdivide CKD patients based on staging, our results are reassuring for physicians treating CKD patients who may be hesitant to undergo TAVR due to concerns about worsening renal function during the procedure. Given the cyclical nature of AS and kidney function in CRS, the progression of CKD could be an early sign of severe AS and an indication that the patient may benefit from TAVR, even before subjective symptoms appear [22]. Further research is needed to better understand the role of early TAVR in asymptomatic patients, particularly those with CRS. Diabetes and hypertension are the most common causes of kidney dysfunction, yet few studies focus on changes in kidney function after TAVR in diabetic and hypertensive populations. Fan et al. [23] studied kidney function after TAVR in diabetic and hypertensive patients, noting that kidney autoregulation is impaired in the presence of these conditions. The pathophysiological mechanisms may relate to the negative influence of diabetes and hypertension on renal autoregulation [24]. Patients with AS and uncontrolled or controlled hypertension may experience irreversible damage due to excessive RAAS activation and decreased afferent arteriolar resistance [25]. A meta-analysis by Mina et al. [26] showed that diabetes is associated with increased AKI and one-year mortality after TAVR. Overall, renal recovery rates are lower in the diabetic and hypertensive population compared to previous studies.

Limitations

Our study was a retrospective analysis conducted at a tertiary referral center in rural Wisconsin, which may not fully represent the experiences in other regions of the world. Additionally, most of our study population was Caucasian, which may limit the generalizability of our findings to other patient populations and racial groups. The CKD patient population was not staged based on eGFR, which could impact the precision of our analysis. We also did not consider the timing of the pre-TAVR workup, including coronary angiogram and CT angiographies, where contrast agents were used. Moreover, the amount of contrast used during the TAVR procedure was not assessed, nor was the approach (e.g., transfemoral, transapical (TA), or transaortic) taken into account. In particular, during the TA-TAVR procedure, the instrumentation of the aorta may cause the dislodgment of calcium plaques and cholesterol emboli to the renal vasculature, potentially resulting in AKI. Since the data were analyzed retrospectively, we cannot rule out the presence of other confounding factors that might have influenced our results.

## Conclusions

Our study results suggest that TAVR is a safe procedure with the potential to either improve or maintain stable renal function. Significant improvements in renal function were observed in both the CKD and non-CKD groups one month after the TAVR procedure. Although a reduction in eGFR was noted post-TAVR, the decline was similar in both groups, indicating that it is independent of having CKD. These findings support the idea that TAVR may have a protective effect on kidney function. Our results can reassure physicians treating CKD patients, as well as those who are hesitant to undergo TAVR due to concerns about worsening renal function. This study is expected to positively influence shared decision-making between CKD patients and the heart team.
